# Partial Hepatectomy Promotes the Development of KRASG12V-Induced Hepatocellular Carcinoma in Zebrafish

**DOI:** 10.3390/cancers16101793

**Published:** 2024-05-08

**Authors:** Mingkai Zhu, Yan Li, Dong Liu, Zhiyuan Gong

**Affiliations:** 1 Department of Biological Sciences, National University of Singapore, Singapore 117543, Singapore; e0437707@u.nus.edu (M.Z.); yan.li@ntu.edu.sg (Y.L.); 2 School of Life Science, Southern University of Science and Technology, Shenzhen 518055, China

**Keywords:** hepatocellular carcinoma, KRAS, partial hepatectomy, zebrafish

## Abstract

**Simple Summary:**

Partial hepatectomy (PH) is a common clinical surgery for managing hepatocellular carcinoma (HCC). A common concern about PH is the high tumor recurrence rate following PH. Previous studies have reported that PH can promote the growth of transplanted HCC in rodents. In the current study, we have used an inducible *kras^G12V^*-driven zebrafish HCC model to investigate the effects of PH on the oncogene-induced de novo HCC development. We found that PH can significantly promote the development of *kras^G12V^*-induced HCC in zebrafish. This enhancing effect could be attributed to the increased oxidative stress and the enhanced deregulation of molecular factors. Our findings may provide references for the future development of novel therapeutic strategies.

**Abstract:**

The purpose of this study was to investigate the effects of PH on the development of oncogenic *kras^G12V^*-induced HCC in zebrafish. The inducible HCC model in Tg(*fabp10a*:rtTA2s-M2; TRE2:EGFP-*kras^G12V^*) zebrafish was used. PH or sham surgery was performed before the induction of oncogenic *kras^G12V^* expression in the livers of transgenic zebrafish. Histological analysis was carried out to determine the progression of HCC and other HCC-associated features including hepatocyte proliferation, extracellular matrix production, and local oxidative stress. The similarity between the process of PH-induced liver regeneration and that of *kras^G12V^*-induced HCC development was further compared by RNA-Seq analysis. The results show that PH promotes the development of *kras^G12V^*-induced HCC in zebrafish possibly through enhancing neutrophil-mediated oxidative stress and promoting the upregulation of *s100a1*, and the downregulation of ribosome biogenesis.

## 1. Introduction

Partial hepatectomy (PH) is a major method for treating early- and mid-stage hepatocellular carcinoma (HCC), especially in regions with a shortage of liver donors and limited medical resources [1,2,3]. It is a well-established procedure to improve HCC patients’ survival, as the 5-year overall survival rate after the surgery reaches 40–75% [1,3,4,5]. Nevertheless, up to 80% of patients who received PH have HCC recurrence within 5 years, which prevents patients from having better prognoses [6].

The process of liver regeneration following PH has been suggested to activate a similar set of regulators with the process of HCC development [7,8]. Previous studies have suggested that PH can create a microenvironment in favor of HCC growth and metastasis with enhanced angiogenesis, cell dedifferentiation, and expression of growth factors [9,10,11]. This idea has been supported by studies using the rodent PH model with transplanted HCC [12,13,14,15,16]. According to these studies, the growth and metastasis of transplanted HCC cells and xenografts were facilitated by PH in rodents. Nevertheless, these studies only simulate the spread of a pre-existing HCC population but not the de novo development of HCC following PH.

Zebrafish have been widely used in cancer research due to their high capacity for forward genetics and phenotype-based, high-throughput screening [17]. Chemical carcinogen-induced zebrafish HCC has shown histology and gene signatures that resemble human HCC, which demonstrate the modeling potential of zebrafish for HCC [17,18]. The EGFR downstream effector RAS is a major oncogenic factor for human cancer, and KRAS mutation is the most frequent isoform among RAS mutations in liver cancer patients [19,20]. KRAS^G12V^ is a hyperactive mutation of KRAS due to its reduced activity of GTPase. To model KRAS mutation-induced HCC with zebrafish, Tg(*fabp10a*:rtTA2s-M2; TRE2:EGFP-*kras^G12V^*) (gz32Tg) (abbreviated as *kras+*), which has the liver-specific expression of *kras^G12V^* under the control of the Tet-on system, has been generated by our lab previously [21]. This inducible HCC model provides an excellent platform for studying the de novo development of HCC because of its rapid and robust tumorigenesis and the reversible induction of HCC.

Tumor recurrence following PH can occur in two forms: spread of remaining tumor cells and the de novo development of new tumors [2]. How PH affects the de novo development of HCC has not been investigated before. In the current study, we have applied the *kras+* zebrafish to investigate the effects of PH on the de novo development of oncogene-induced HCC. We have found that *kras^G12V^*-induced HCC developed faster in the PH-treated livers than in the control livers. Furthermore, phenotypes associated with HCC development such as hepatocyte proliferation, production of extracellular matrix, recruitment of neutrophils, and local oxidative stresses were enhanced by PH. Transcriptomic analysis revealed the overlap of several deregulated genes between the PH livers and the *kras+* livers. Both PH-induced liver regeneration and *kras^G12V^*-induced HCC development caused significant inhibition of ribosome biogenesis in the zebrafish livers. Together, PH can promote the development of *kras^G12V^*-induced HCC in zebrafish, probably through the enhanced oxidative stresses and inhibited ribosome biogenesis in livers.

## 2. Materials and Methods

### 2.1. Zebrafish Husbandry

The maintenance of the zebrafish used in the current study followed the Institutional Animal Care and Use Committee guidelines from the Southern University of Science and Technology (SUSTech), China, and the National University of Singapore (NUS). The transgenic zebrafish used in the current study include Tg(*fabp10a*:DsRed; *ela3l*:EGFP) (gz15Tg) [22], Tg(*lyz*:DsRed) (nz50Tg) with DsRed-labelled neutrophils [23]; Tg(*fabp10a*:rtTA2s-M2; TRE2:EGFP-*kras^G12V^*) (gz32Tg) with the inducible liver-specific expression of oncogenic *kras^G12V^* in a Tet-On system [21]; and double-transgenic zebrafish Tg(*fabp10a*: rtTA2s-M2; TRE2: EGFP-*kras^G12V^*; *lyz*: DsRed) generated by crossing Tg(*fabp10a*:rtTA2s-M2; TRE2:EGFP-*kras^G12V^*) with Tg(*lyz*:DsRed), which were designated as Lipan, *lyz+*, *kras+,* and *kras/lyz+*, respectively.

### 2.2. Zebrafish Partial Hepatectomy

Adult male zebrafish (>12 weeks old) were used in the experiments of partial hepatectomy (PH). The procedure of performing PH on zebrafish was derived from the published protocol [24]. In general, after anesthesia with 150 mg/L of buffered tricaine (MS-222), a 2–3 mm incision was made on the zebrafish abdomen with a micro-stab knife and precise scissors to expose the liver. Subsequently, the ventral lobe of the liver was removed with tweezers, resulting in approximately 30% of the liver being resected. Sham surgery was performed as a control by making an incision on the abdomen without removing the liver tissue.

### 2.3. Induction of the Transgenic Oncogene Expression

To induce the expression of transgenic *kras^G12V^* in the liver of *kras+* zebrafish, adult and larvae *kras+* zebrafish were exposed to 20 mg/L of doxycycline (Dox) (D9891; Sigma-Aldrich, St. Louis, MO, USA). For larvae exposure, Dox treatment began at 3 dpf for 48 h. For adult exposure, Dox treatment was continued for up to 7 days. During Dox treatment, the zebrafish were kept in dim light to prevent light-sensitive degradation of Dox.

### 2.4. RNA Extraction and RT-qPCR

The total RNA extraction from tissues and embryos was performed using TRIzol reagent (Invitrogen, Waltham, MA, USA), followed by reverse transcription using a Transcriptor First Strand cDNA Synthesis Kit (Roche, Basel, Switzerland). The synthesized cDNA was used for real-time quantitative PCR (RT-qPCR) with SsoAdvanced Universal Supermixes (Bio-Rad Laboratories, Hercules, CA, USA), GoTaq qPCR Master Mix (Promega, Madison, WI, USA) in CFX96 Touch Real-Time PCR System (Bio-Rad Laboratories), and ABI 7500 Fast Dx Real-Time PCR Instrument (Thermo Fisher Scientific, Waltham, MA, USA), respectively. Genes of interest were amplified by 40 cycles using a standard three-step protocol (95 °C, 20 s; 65 °C, 15 s; 72 °C, 30 s). All primers used in RT-qPCR are listed in Table S1.

### 2.5. RNA-Seq and Bioinformatic Analysis

The RNAs used for RNA-Seq were extracted from the liver tissues with TRIzol reagent. For the RNAs extracted from PH/sham surgery-treated WT zebrafish, library preparation and Illumina sequencing were conducted by Novogen, Hong Kong. Each sample used for RNA-Seq contained RNAs from three individual zebrafish livers, and each biological group contained two pooled samples. For the RNAs extracted from *kras+* and WT zebrafish after Dox treatment, library preparation and Illumina sequencing were conducted by Metware Biotechnology, New Zealand. Each sample used for RNA-Seq contained RNAs from two individual zebrafish livers, and each biological group contained three pooled samples. After sequencing, the reads were mapped to zebrafish reference genome assembly GRCz11 (https://www.ncbi.nlm.nih.gov/assembly/GCF_000002035.6/, accessed on 5 March 2023) with TopHat2 (http://ccb.jhu.edu/software/tophat/index.shtml, accessed on 4 January 2021). The raw counts of sequences mapped to each gene were quantified using StringTie (https://ccb.jhu.edu/software/stringtie/, accessed on 4 January 2021), followed by differential expression analysis using DESeq2 [25]. The criteria of significantly deregulated genes were adjusted *p*-value (padj) ≤ 0.05 and fold change (FC) ≥ 2. To identify enriched pathways, pre-ranked gene set enrichment analysis (GSEA) was performed using the FGSEA package and clusterProfiler package [26,27]. The Hallmark pathway gene sets and KEGG pathway gene sets used for GSEA were downloaded from the Molecular Signature Database and the KEGG database, respectively [28,29]. The differentially expressed genes were ranked by their values of Wald statistic generated from DESeq2.

### 2.6. Morpholino Knockdown

For *s100a1* knockdown, MO-*s100a1*-ATG (5′-GTGACGACAGCTTGAAAAGATGTT-3′) (Gene Tools, Philomath, OR, USA) was synthesized and applied. Aliquots of morpholino (50 nM) were injected into zebrafish embryos at the one-cell stage. The effect of MO-*s100a1*-ATG on S100A1 expression was validated in 1-dpf zebrafish embryos with western blotting using the Rabbit-anti-βactin antibody (bs-0061R, Bioss Antibodies, Woburn, MA, USA) (1:5000) and the Rabbit-anti-S100A1 antibody (50266-RP02; Sino Biological, Beijing, China) (1:1000). The total protein extraction from zebrafish embryos and western blotting were conducted as previously described [30].

### 2.7. Histological Analyses

Adult zebrafish liver and gut tissues were harvested together and fixed in 10% formalin and 4% paraformaldehyde (PFA) in PBS overnight for subsequent paraffin embedding and cryo-embedding, respectively. Paraffin-embedded samples were sectioned at 5 μm with a microtome, and frozen samples were sectioned at 8 μm with a cryostat. All frozen samples were used for immunofluorescence (IF) staining, while paraffin-embedded samples were primarily used for hematoxylin and eosin (H&E) staining and immunohistochemistry (IHC) staining. For IHC staining, sections were incubated with diluted primary antibodies overnight at 4 °C, followed by incubation with a horseradish peroxidase (HRP)-conjugated secondary antibody and color development with the DAKO Real Envision Detection System (K500711; Agilent, Santa Clara, CA, USA). For IF staining, the sections were incubated with diluted primary antibodies overnight at 4 °C, followed by incubation with diluted fluorescent-dye-conjugated secondary antibodies for 2 h at room temperature. The antibodies used in this study and their dilution ratio are Mouse anti-PCNA (sc-56; Santa Cruz) (1:200), Rabbit anti-HNF4α (ab201460; Abcam, Cambridge, UK) (1:2000), Rabbit anti-HIF1α (ab114977; Abcam) (1:200), Goat anti-rabbit IgG Alexa Fluor™ 488 (A11034; Invitrogen) (1:500), and Goat anti-mouse IgG Alexa Fluor™ 568 (A11031; Invitrogen) (1:500). During immunostaining, the tissue sections received heat-mediated antigen-retrieval by 95 °C water baths in citrate buffer (C9999; Sigma-Aldrich). For H&E staining, the rehydrated sections were stained with Mayer’s hematoxylin (Vector Laboratories, Newark, CA, USA) and eosin (Sigma-Aldrich). The classification of liver histology was conducted as previously described [31].

### 2.8. Photography and Image Analysis

Side views of dissected adult zebrafish were photographed individually with a stereo fluorescence microscope (SZX16; Olympus, Tokyo, Japan). Larvae photography was performed with the ZEISS Axio Imager 2 light microscope (Carl Zeiss AG, Oberkochen, Germany) after immobilizing the larvae in 3% methylcellulose. For IHC and H&E staining, theimaging was conducted using the ZEISS Axio Imager 2 light microscope and the NanoZoomer S60 digital slide scanner (Hamamatsu Photonics, Shizuoka, Japan). For imaging IHC and H&E staining, at least two high-power fields of view per sample were captured and analyzed. Tissue sections of IF staining were imaged with the ZEISS LSM 900 confocal laser scanning microscope (Carl Zeiss AG, Oberkochen, Germany). For imaging IF staining, at least three high-power fields of view per sample were captured and analyzed. All image analyses were performed with ImageJ [32].

### 2.9. Statistical Analysis

For statistical analysis, Prism 8.0 (GraphPad, San Diego, CA, USA) was applied. All statistical significances in the current study were determined by a two-tailed unpaired student *t*-test unless stated otherwise.

## 3. Results

### 3.1. PH Promotes kras^G12V^-Induced HCC Development in Zebrafish

**Figure 1:**
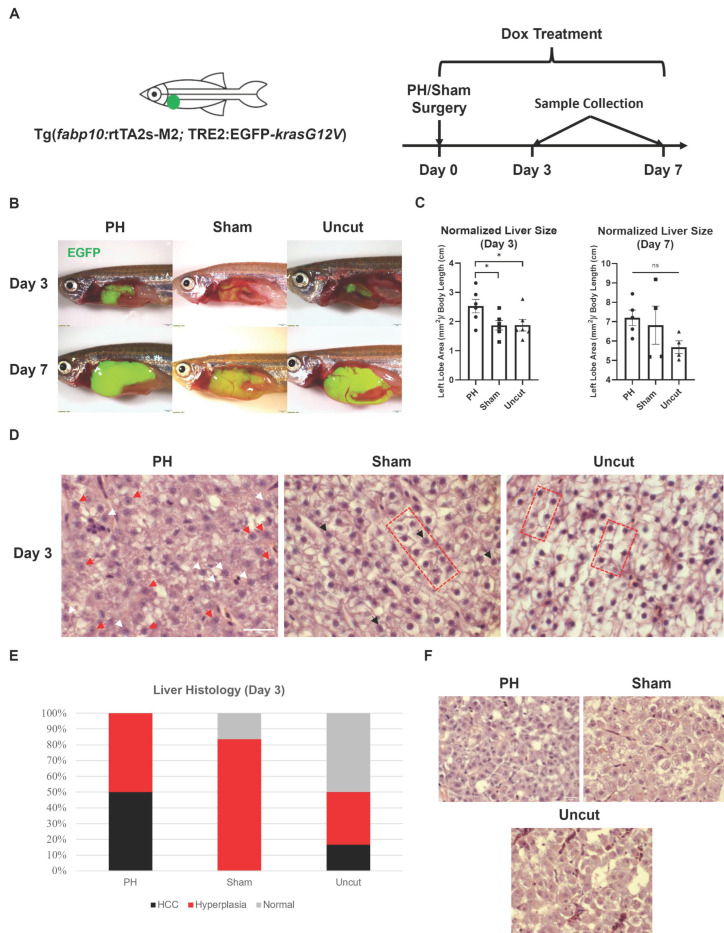
Effects of PH on the *kras^G12V^*-induced development of HCC in zebrafish. (**A**) Schedule of surgeries and Dox treatment in the experiment. Right after PH or sham surgery, *kras+* male zebrafish were exposed to 20 mg/L Dox to induce the overexpression of oncogene kras^G12V^ in the liver. (**B**) Representative photos of PH/sham surgery-treated *kras+* zebrafish 3 days and 7 days after Dox induction. (**C**) Measurement and comparison of the 2D area of the *kras+* livers in mm^2^ based on (**B**) after being normalized with the body length in cm (*n* ≥ 4). Circles, squares, and triangles indicate values of individual samples. (**D**) Representative H&E staining images of the PH/sham surgery-treated *kras+* zebrafish livers 3 days after Dox induction (*n* = 6). Examples of morphology associated with HCC and hyperplasia are indicated with symbols. Red arrowheads: irregular nuclei with prominent nucleoli; white arrowheads: nuclei with multiple nucleoli; black arrowheads: enlarged nuclei with prominent nucleoli; red bracket: two-cell hepatocyte plate structures. (**E**) Quantification of HCC histology observed in *kras+* zebrafish from each treatment group on Day 3 based on (**D**). (**F**) Representative H&E staining images of the PH/sham surgery-treated *kras+* zebrafish livers 7 days after Dox induction (*n* = 6). Scale Bar = (**B**) 1 mm and (**D**,**F**) 20 μm. ns *p* > 0.05, * *p* ≤ 0.05.

To investigate the effect of PH on the *kras^G12V^*-induced development of HCC in zebrafish, we performed PH and sham surgery on *kras+* zebrafish before inducing the liver-specific expression of *kras^G12V^* with continuous Dox treatment (Figure 1A). Only male zebrafish were used in the current study because previous studies have demonstrated that HCC development in zebrafish is male-biased [33,34]. After 3 days of Dox induction, the PH zebrafish developed significantly larger livers than the sham and uncut zebrafish (Figure 1B,C). By Day 7, all zebrafish had developed excessive livers, which overshadowed the effect of PH on liver growth. These results indicate that PH facilitated *kras^G12V^*-induced hepatomegaly. Subsequently, we evaluated the histology of the *kras+* liver sections after H&E staining. Based on liver histology, we found more HCC fish in the PH group than in the two control groups on Day 3 (Figure 1D,E). Normal liver histology was not observed in the PH group on Day 3, indicating that all zebrafish in that group had either developed HCC or had been at the pre-stage of HCC development (liver hyperplasia). The sham group had more zebrafish with liver hyperplasia than the uncut group, which could be attributed to the inflammatory effects caused by the wound. Generally, PH-treated zebrafish have more advanced HCC progressions than the control zebrafish. By Day 7, all *kras+* zebrafish had the liver histology of HCC due to the robust effect of oncogene overexpression across the whole liver (Figure 1F). Therefore, the oncogene *kras^G12V^*-induced development of HCC in zebrafish can be facilitated by PH.

### 3.2. PH Enhanced the HCC-Associated Features in Kras+ Zebrafish

**Figure 2:**
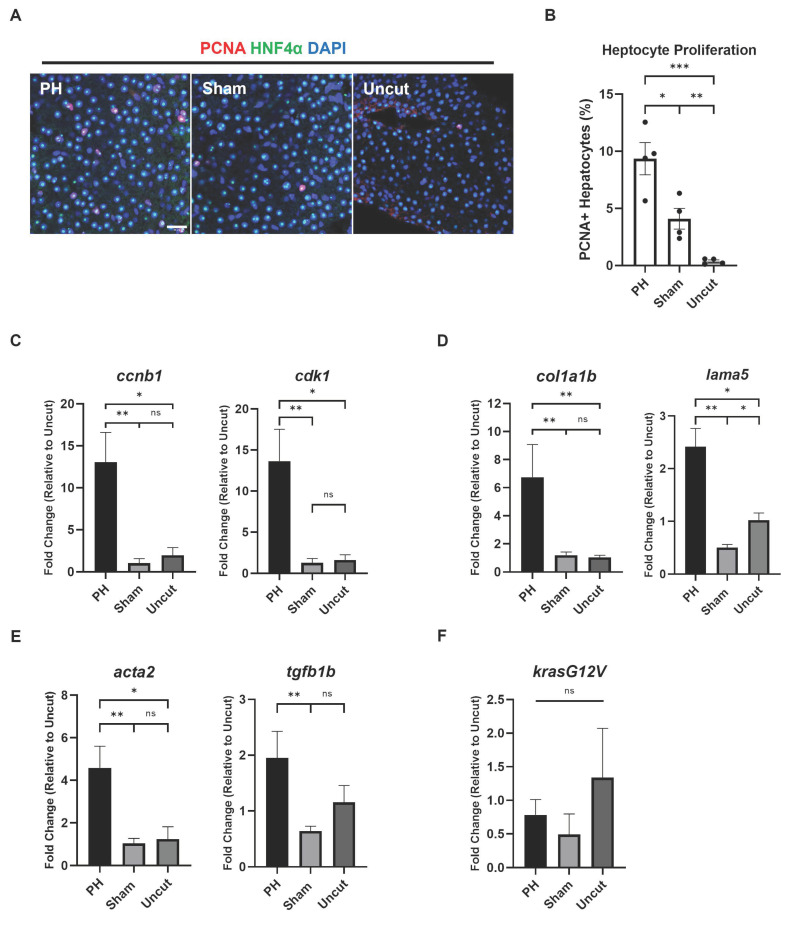
Effects of PH on the progress of HCC-associated characteristics in *kras+* zebrafish. (**A**) IF staining of PCNA and HNF4α in the PH/sham surgery-treated *kras+* zebrafish liver 3 days after Dox induction. (**B**) Quantification of PCNA and HNF4α-double positive proliferating hepatocytes based on (**A**) (*n* = 4). Circles indicate values of individual samples. (**C**–**E**) Expression of (**C**) *ccnb1* and *cdk1,* (**D**) *col1a1b, lama5,* (**E**) *acta2* and *tgfb1b* in the PH/sham surgery-treated *kras+* zebrafish livers 3 days after Dox induction as determined by RT-qPCR (*n* = 4). (**F**) Expression of *kras^G12V^* in the PH/sham surgery-treated *kras+* zebrafish livers 3 days after Dox induction as determined by RT-qPCR (*n* = 3). Scale Bar = 20 μm. ns *p* > 0.05, * *p* ≤ 0.05, ** *p* ≤ 0.01, *** *p* ≤ 0.001.

Because PH facilitated the histopathological transformation of the *kras+* zebrafish livers, we speculated that PH may also promote other HCC-associated features. The development of *kras^G12V^*-induced HCC in zebrafish is associated with robust cell proliferation, which has been demonstrated by previous studies [33,34]. To examine the effect of PH on the proliferation of hepatocytes during the *kras^G12V^*-induced HCC development, IF staining of the proliferation marker PCNA and the hepatocyte marker HNF4α was conducted. Based on IF imaging, the PH-treated *kras+* zebrafish had significantly higher levels of hepatocyte proliferation than the control groups after 3 days of Dox induction, which explained the enhanced liver expansion in the PH group (Figure 2A,B). Thus, PH can promote hepatocyte proliferation during the *kras^G12V^*-induced zebrafish’s HCC development.

We then examined the effects of PH on the expression of HCC-associated genes by RT-qPCR in the *kras+* zebrafish livers from different treatment groups after 3 days of Dox induction. Consistent with the enhanced cell proliferation, the liver expression of cell cycle-related genes, including *ccnb1* and *cdk1*, was promoted significantly by PH (Figure 2C). Extracellular matrix (ECM) accumulation in the liver can contribute to tumor metastasis during HCC development [35]. In our experiments, the expression of genes encoding ECM, including *col1a1b* and *lama5*, was significantly increased by PH in the *kras+* livers (Figure 2D). Furthermore, the genes that contribute to ECM production and liver fibrosis, including myofibroblast marker *acta2* and cytokine *tgfb1b*, were also significantly upregulated by PH in the *kras+* livers (Figure 2E). To rule out the possibility of discrepancy in the oncogene expression levels among the different groups, we examined the expression levels of *kras^G12V^* in the *kras+* livers and found no significant differences among the three groups (Figure 2F).

### 3.3. PH Enhances the Oxidative Stress and Neutrophil Recruitment in the Kras+ Zebrafish Liver

**Figure 3:**
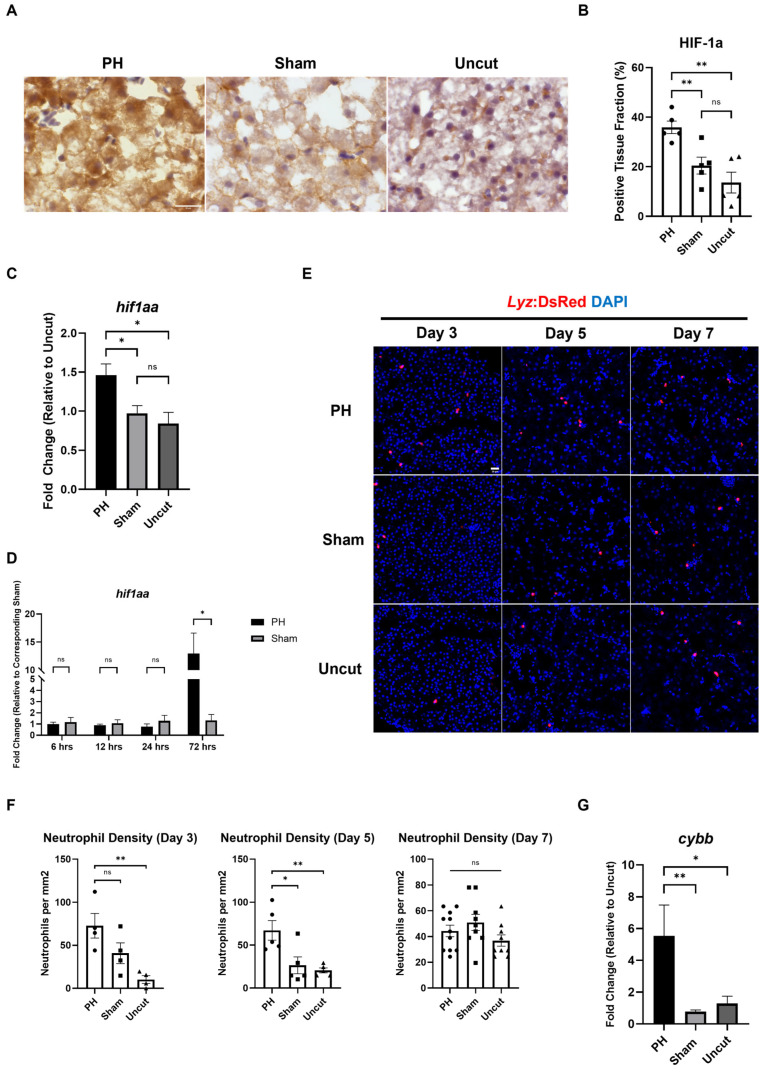
Effects of PH on oxidative stress and neutrophil activity in the *kras+* zebrafish liver during the *kras^G12V^*-induced development of HCC. (**A**) IHC staining of HIF1α in PH/sham surgery-treated *kras+* livers on Day 5. (**B**) Quantification of HIF1α-positive liver tissue based on (**A**) (*n* = 5). The HIFα positive fraction was determined by measuring the DAB-stained area versus the total tissue area with imageJ plugin color deconvolution. (**C**) Expression of *hif1aa* in PH/sham surgery-treated *kras+* livers on Day 3 as determined by RT-qPCR (*n* = 5). (**D**) Expression of *hif1aa* in WT male livers within 72 h after PH/sham surgery as determined by RT-qPCR (*n* = 3). (**E**) Fluorescence images of PH/sham surgery-treated kras/*lyz+* livers following Dox induction. (**F**) Quantification of DsRed+ neutrophils in the liver based on (**E**) (*n* ≥ 4). (**G**) Expression of *cybb* in PH/sham surgery-treated kras/*lyz+* livers on Day 3 as determined by RT-qPCR (*n* = 4). Scale Bar = 20 μm. Circles, squares, and triangles in (**B**,**F**) indicate values of individual samples. ns *p* > 0.05, * *p* ≤ 0.05, ** *p* ≤ 0.01.

The reactive oxygen species (ROS)-induced oxidative stress has been reported to correlate with the development of HCC [36]. Based on the results of the IHC staining, we found that the protein level of hypoxia-inducible factor 1-alpha (HIF1α) in the *kras+* zebrafish liver was significantly higher in the PH group than in the sham group on Day 5 (Figure 3A,B). In addition to that, the expression of the *hif1aa* gene, which encodes HIF1α, was upregulated significantly in the PH-treated *kras*+ livers compared with the control *kras+* livers on Day 3 (Figure 3C). To confirm whether PH alone can induce HIF1α upregulation, we examined the expression of *hif1aa* in WT male zebrafish livers following PH/sham surgery. As shown in Figure 3D, the expression of *hif1aa* was not changed by 24 h post-PH but upregulated significantly by 72 h post-PH. These results show that PH could enhance oxidative stress in the *kras+* liver. As a main source of ROS, tumor-associated neutrophils (TANs) have been reported to play essential roles in liver regeneration and HCC development [37,38]. To investigate the effect of PH on the recruitment of neutrophils during HCC development, we employed the *kras/lyz+* zebrafish. Based on IF imaging results, PH significantly increased the number of neutrophils in the *kras/lyz+* liver after 5 days of Dox induction (Figure 3E,F). Neutrophils produce ROS mainly through activating NADPH oxidase 2 (NOX2) [39]. In our experiments, the expression of *cybb*, which encodes NOX2, in the *kras/lyz+* liver was increased significantly by PH 3 days after Dox induction (Figure 3G). Therefore, the enhanced recruitment of neutrophils in the PH *kras/lyz+* liver may promote ROS production, which contributes to the development of HCC in zebrafish.

### 3.4. Comparison of Liver Transcriptomic Regulation between PH-Induced Liver Regeneration and Kras^G12V^-Induced Hepatocellular Carcinogenesis

**Figure 4:**
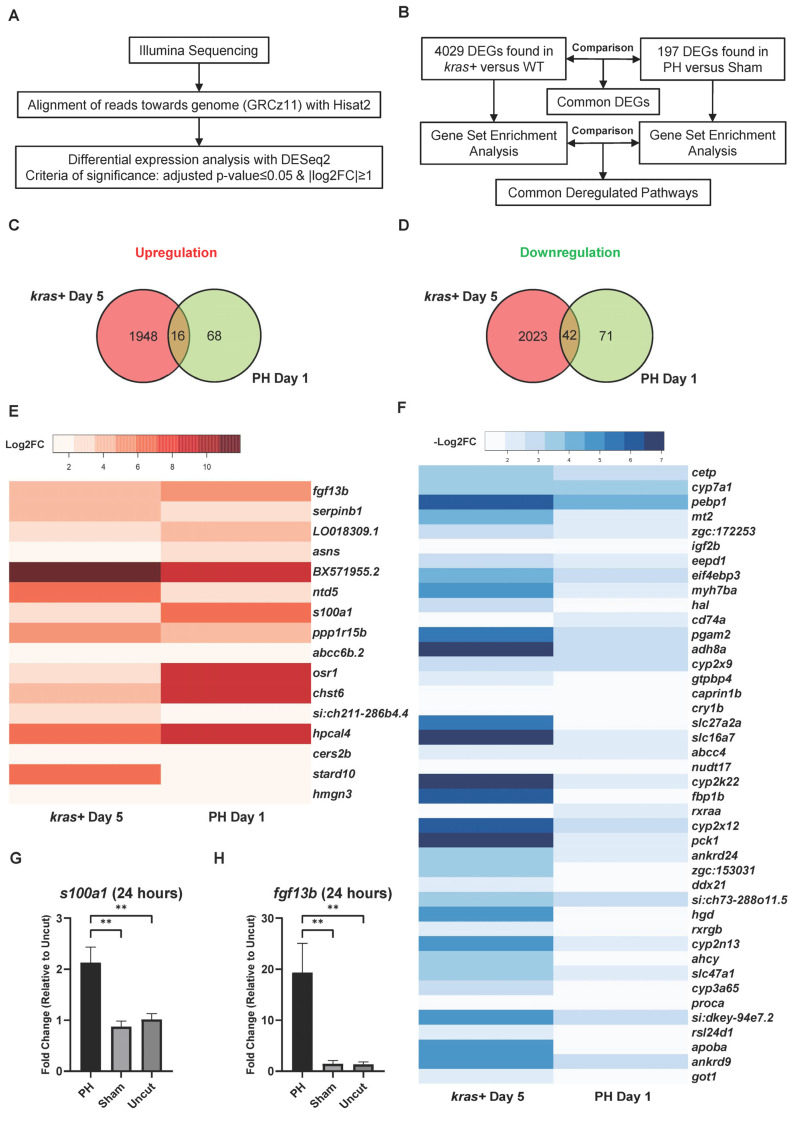
Comparison of DEGs during *kras^G12V^*-induced HCC development and PH-induced liver regeneration in zebrafish. (**A**) Workflow of the differential expression analysis based on the RNA-Seq reads. (**B**) Experimental design for comparing the transcriptomic changes in the *kras+* livers with those in the PH livers with the quantification of total DEGs. (**C**,**D**) Venn diagrams showing the overlaps of (**C**) upregulated genes and (**D**) downregulated genes between the *kras+* male liver on Day 5 and the PH male liver on Day 1. (**E**,**F**) Heatmaps of genes that were significantly (**A**) upregulated and (**B**) downregulated in both the *kras+* liver on Day 5 and the PH liver on Day 1 based RNA-seq data. (**G**,**H**) Expression of (**G**) *fgf13b* and (**H**) *s100a1* in the *kras+* male livers 24 h after PH/sham surgery as determined by RT-qPCR (*n* = 4). ns *p* > 0.05, ** *p* ≤ 0.01.

To investigate the mechanism underlying the promoting effect of PH on the development of *kras^G12V^*-induced zebrafish HCC, we performed RNA-Seq with the *kras*+ and WT male zebrafish livers on Day 5 after Dox induction and determined the differential expressed genes (DEGs) of the *kras+* livers versus the WT livers (Figure 4A). Similarly, RNA-Seq was also performed using the WT male zebrafish livers on Day 1 after PH/sham surgery, and the DEGs in the PH livers compared with the sham livers were determined. In total, 4029 DEGs were found in the *kras+* livers, while 197 DEGs were found in the PH livers (Figure 4B). We then compared the DEGs induced by HCC development with those induced by PH. Sixteen common upregulated and 42 common downregulated genes were found in the *kras*+ livers and PH livers (Figure 4C,D). Because previous studies have suggested that PH could promote HCC development through additive effects, we focused on the overlap of DEGs between the PH livers and the *kras+* livers (Figure 4E,F) (Table S2) [7,8]. *fgf13b and s100a1* were significantly upregulated in both the *kras*+ livers and the PH livers. By RT-qPCR, we confirmed that PH could further upregulate the expression of *fgf13b and s100a1* during *kras^G12V^*-induced HCC development at 24 h post-PH (Figure 4G,H). Both FGF13 and S100A1 have been suggested to promote cancer cell growth and survival by previous studies [40,41]. Thus, the enhanced expression of *fgf13b* and *s100a1* in the PH-treated livers may contribute to the promoting effects of PH on HCC development. Curiously, the *kras+* livers and the PH livers showed an overlap in the significant downregulation of *rsl24d1*, which is crucial to the biogenesis of the 60S large ribosome subunit [42]. Apart from some genes of interest found in the common DEGs between the PH livers and the *kras+* livers, the majority of DEGs in the PH livers were not found among the DEGs in the *kras+* livres (Table S3). Therefore, it is possible that PH can also affect HCC progression through mechanisms completely different from *kras^G12V^*-induced tumorigenesis.

**Figure 5:**
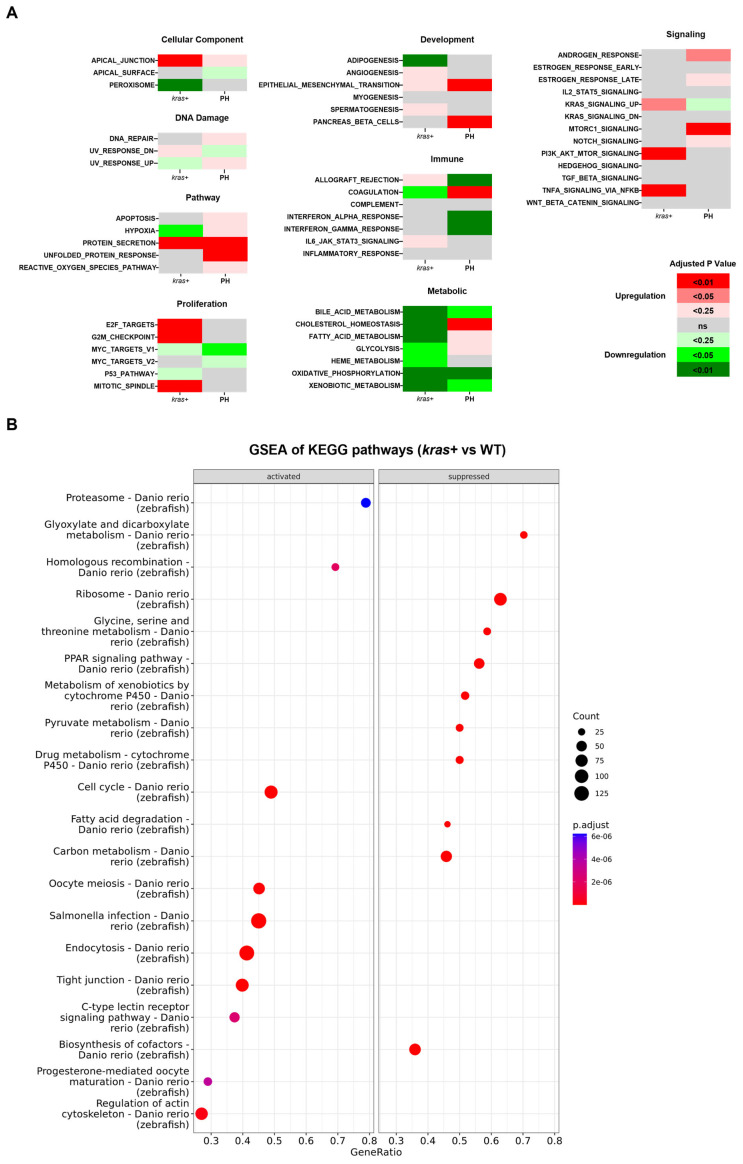

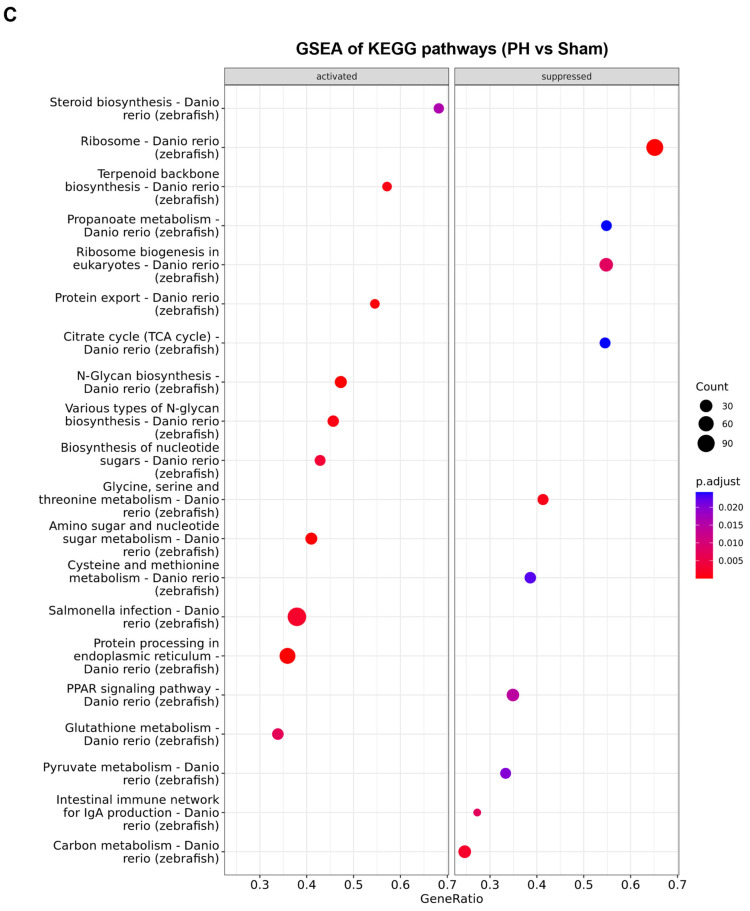
Comparison of changes in biological functions and pathways between the *kras+* zebrafish liver and the PH liver. (**A**) Comparison between the changes in the activity of Hallmark gene sets in the *kras+* liver on Day 5 following Dox induction and those in the PH livers on Day 1 following PH based on the GESA of Hallmark gene sets. The color key indicates the direction of changes (red: upregulation; green: downregulation) and significance (adjusted *p*-value). (**B**,**C**) GESA of KEGG pathway in (**B**) *kras+* livers on Day 5 following Dox induction and (**C**) PH livers on Day 1 following PH. The size of the dots indicates the number of leading-edge genes in the corresponding gene set. The color key indicates significance in adjusted *p*-value. Gene Ratio is the ratio of leading-edge genes versus all genes in the gene set.

**Figure 6:**
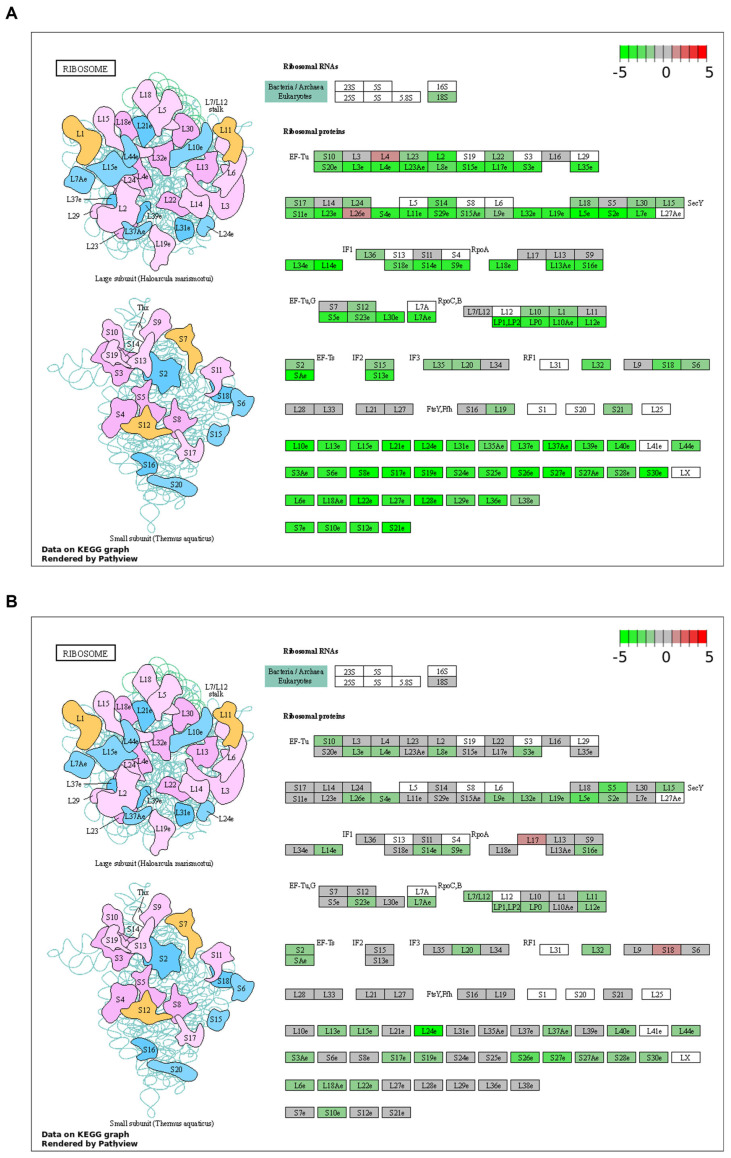
Regulation of ribosome protein genes and ribosomal RNAs during *kras^G12V^*-induced zebrafish HCC development and PH-induced zebrafish liver regeneration. (**A**) Regulation of genes under the KEGG ribosome pathway in *kras+* livers after 5 days of Dox induction. (**B**) Regulation of genes under the KEGG ribosome pathway in PH livers at 1 day post-PH.

By gene set enrichment analysis (GSEA) of the Hallmark gene sets and KEGG pathways, we found that the deregulation of biological functions and pathways caused by *kras^G12V^* overexpression shared little similarity with those caused by PH-induced liver regeneration (Figure 5A–C). These results were consistent with the small overlaps of DEGs between the *kras+* liver and the PH liver. The GSEA of Hallmark gene sets indicated an overlap in the activation of epithelial to mesenchymal transition between the *kras+* liver and the PH liver, which explained the enhanced production of ECM in the *kras+* livers after PH. Apart from that, based on the GSEA of KEGG pathways, both the *kras+* liver and the PH liver had a significant downregulation in the ribosome pathway. As shown in Figure 6A,B, the expression of several ribosomal proteins such as L5e, L24e, S26e, and S27e was reduced in both the *kras+* liver and the PH liver, suggesting that both PH and *kras^G12V^* overexpression caused a general inhibition on ribosome biogenesis in the zebrafish liver. FGF13-mediated repression on ribosome biogenesis can reduce cell death caused by acute oncogene overexpression and promote cancer cell growth in vitro [40]. Thus, it is possible that PH promoted *kras^G12V^*-induced HCC development in zebrafish by an additive effect on the downregulation of ribosome biogenesis.

**Figure 7:**
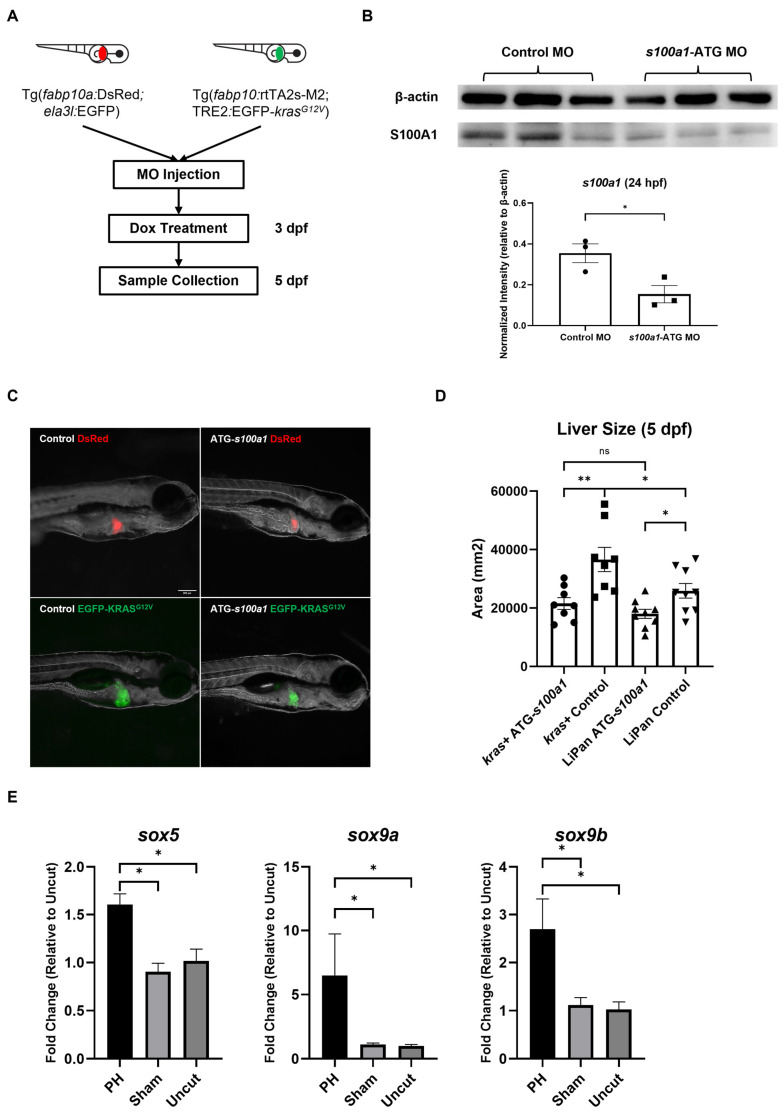
The effect of *s100a1* knockdown on the development of *kras^G12V^*-induced HCC in zebrafish larvae. (**A**) Experiment design of *s100a1* knockdown and oncogene induction in Lipan larvae and *kras+* larvae. (**B**) Western blot of S100A1with the total protein extracted from MO-injected zebrafish embryos at 24 hpf. β-actin served as a loading control. Each protein sample was pooled from 15 zebrafish embryos. The quantification of the intensity of S100A1normalized by the intensity of β-actin was at the lower panel. The uncropped image of blots is shown in Figure S1. (**C**) Fluorescence images of morpholino-treated Lipan and kras+ larvae after 48 h of Dox treatment. (**D**) Measurement of liver size based on the fluorescence in (**B**) (*n* ≥ 8). Circles, squares, and triangles indicate values of individual samples (**E**) Expression of *sox5, sox9a,* and *sox9b* in the *kras+* livers at 24 h after PH/sham surgery as determined by RT-qPCR (*n* = 3). Scale Bar = 200 μm. ns *p* > 0.05, * *p* ≤ 0.05, ** *p* ≤ 0.01.

With regard to S100A1, it has been suggested that S100A1regulates the Hippo-YAP-signaling pathway, which is an oncogene suppressor, in HCC cells [41]. To test whether S100A1plays a role in *kras^G12V^*-induced zebrafish’s HCC development, we completed knockdown of *s100a1* in *kras+* larvae and LiPan larvae with the morpholino (MO) before Dox treatment (Figure 7A). The effectiveness of the morpholino targeting the start codon of *s100a1* (*s100a1*-ATG) was verified with western blot analysis (Figure 7B). As shown in Figure 7C,D, *s100a1* knockdown abolished the hepatomegaly caused by *kras^G12V^* overexpression after 48 h of Dox induction in *kras+* larvae. Thus, *s100a1* was required for the *kras^G12V^*-induced tumor growth, and the enhanced *s100a1* expression in the PH livers may contribute to HCC development in *kras+* zebrafish. Notably, the *s100a1*-ATG MO-treated LiPan larvae showed significantly smaller liver sizes than the control MO-treated larvae, indicating that *s100a1* knockdown can also compromise normal liver growth.

Sox family protein Sox9 has been reported to activate the expression of *s100a1*, and this regulatory function of Sox9 can be enhanced by Sox5 [43]. To determine the mechanism underlying the upregulation of *s100a1* in the PH-treated *kras+* livers, we examined the expression of *sox5, sox9a,* and *sox9b* in the *kras+* male zebrafish livers at 24 h post-PH by RT-qPCR. As shown in Figure 7E, PH significantly enhanced the expression of *sox5*, *sox9a*, and *sox9b* in the *kras+* livers. Thus, the enhanced *s100a1* expression in the PH-treated *kras+* livers could be due to the upregulation of *sox5* and *sox9* induced by PH.

## 4. Discussion

As mentioned earlier, the recurrence of HCC following PH can be classified into two types: the expansion of pre-existing tumors and the de novo development of new tumors [2]. According to previous studies, transplanted HCC cells and xenografts in rodents have shown enhanced growth and metastasis after PH [12,13,14,15,16]. With the inducible HCC zebrafish model driven by *kras^G12V^*, the current study was the first one to investigate the effects of PH on the oncogene-induced de novo development of HCC. We found that PH promoted HCC development indicated by the enhanced liver expansion and facilitated the pathological transformation of liver histology 3 days after the induction of oncogene overexpression. The recruitment of neutrophils and the local oxidative stress in the *kras+* livers were also enhanced by PH. Neutrophils-derived ROS has been suggested to promote tumor cell growth in vitro and hepatocarcinogenesis in rodents [44,45]. Thus, the enhanced HCC development in the PH-treated *kras+* zebrafish could be attributed to the enhanced neutrophil recruitment and local oxidative stresses in their livers.

In addition to the facilitated development of HCC-associated histopathology, PH enhanced hepatocyte proliferation and ECM production in the *kras^G12V^*-induced zebrafish HCC. Sustaining cell proliferation is the fundamental feature of cancers because it drives the growth of tumors [46]. Thus, the enhanced expansion of livers in the PH-treated *kras+* zebrafish can be attributed to their enhanced hepatocyte proliferation. Disorganized ECM contributes to the formation of the tumor microenvironment. Excessive ECM deposition in the liver could lead to liver fibrosis and cirrhosis, which predispose to HCC and promote tumor growth and metastasis by inducing inflammatory factors, promoting abnormal vasculature, and increasing tissue rigidity [35]. In the current study, the PH-treated *kras+* zebrafish livers have shown enhanced ECM gene expressions, which could contribute to the speed of tumorigenesis. Consistent with our results, the promoting effects of PH on hepatocyte proliferation and ECM production have also been reported in rodent HCC models [13,15,16].

Previous studies have summarized a sizable set of genes and signaling pathways that are shared between HCC development and liver regeneration [7,8]. Unexpectedly, our RNA-Seq results showed a very limited pool of genes that were deregulated in the same direction by both *kras^G12V^* overexpression and PH-induced liver regeneration. This could be due to the fact that the time points of sampling the PH/sham zebrafish were too late to catch more significant changes. In addition, because the comparison was made between two independent experiments conducted with different batches of zebrafish, the differences in their genetic backgrounds may interfere with the results. Among the several genes that were significantly upregulated in both *kras+* livers and PH livers, *fgf13b* has the potential to contribute to the enhanced HCC development in *kras+* zebrafish after PH. FGF13 plays dual roles in cell proliferation depending on the types of cells and tissues, as reported by previous studies [47,48]. In tumorigenesis, *fgf13* was found to be upregulated in lung adenocarcinoma patients and to be essential to the survival of cancer cells in vitro [40]. Another candidate gene that was significantly upregulated by both *kras^G12V^*-induced HCC development and PH-induced liver regeneration is *s100a1*. The upregulation of *s100a1* has been found in various types of tumors including HCC in the clinic, and a high S100A1level is correlated with poor outcomes for patients [41,49,50]. It was demonstrated that S100A1promoted HCC cell proliferation in vitro by promoting YAP activation [41]. Thus, it is possible that PH enhances YAP activity during HCC development by further enhancing the expression of *s100a1*.

Based on the GSEA of the KEGG pathway, we found that the ribosome pathway was downregulated in both the *kras+* livers and the PH livers. The downregulation of the ribosome pathway in the *kras+* livers is consistent with the previous data our lab obtained from the *kras^G12V^*-driven HCC and the *xmrk*-driven HCC in zebrafish [51]. These observations seem counterintuitive because the intense cell proliferation during HCC development should require ribosome-mediated protein synthesis. Several ribosomal proteins have been reported to be overexpressed in HCC and contribute to tumorigenesis [52]. However, they are usually involved in specific oncogenic pathways rather than merely mediating protein synthesis universally. Moreover, a previous study analyzing RNA-Seq data from 372 human HCC tissues has shown that most genes in the KEGG ribosome pathway were downregulated, which is similar to our data from the current study [53]. In the C-myc-induced mouse HCC model, the downregulation of ribosomal proteins and rRNAs was observed in the livers during the early formation of HCC but not in the advanced HCC, suggesting that ribosome biogenesis was not regulated uniformly across different stages of tumorigenesis [54]. The downregulation of the ribosome pathway in the *kras+* livers and PH livers occurred simultaneously with the upregulation of *fgf13b*. FGF13 has been found to promote cancer cell survival and growth by repressing ribosome biogenesis and restricting protein synthesis to reduce endoplasmic reticulum stress [40]. It was not directly upregulated by acute overexpression of the oncogene but was increased gradually during the oncogene-induced neoplastic transformation of cells. Similarly, the downregulation of rRNAs and ribosomal proteins was observed in C-myc-induced mouse HCC only after 7 days of overexpression of the oncogene [54]. Thus, the upregulation of *fgf13* and the downregulation of ribosome biogenesis could occur temporally in response to endoplasmic reticulum stress during the formation of tumors to enhance the survivability of cancer cells. Enhanced *fgf13b* expression in the PH-treated *kras+* livers may contribute to the facilitated HCC development by mediating the downregulation of ribosome biogenesis. In the clinic, the protein synthesis inhibitor sorafenib is commonly applied to manage HCC. The possibility of repressed protein synthesis promoting HCC development at certain stages shows a potential need to further optimize the clinical application of protein synthesis inhibitors.

As for the limitations of the current study, although the EGFR downstream effector RAS is a major proto-oncogene for human cancer, the RAS mutation is not very prevalent in patients with liver cancers, with 7% of patients having KRAS mutations and 4% of patients having HRAS mutations [20]. It is unclear whether the findings from the current study can be applied to HCC induced by other types of oncogenes. However, up to 50% of HCC patients have been reported to have RAS activation, and RAS is a common therapeutic target for HCC in the clinic [55]. Thus, the *kras+* zebrafish HCC model used in our study has the potential to represent the situation of a substantial category of HCC. In addition, the *kras+* zebrafish has the ectopic expression of *kras^G12V^* across the whole liver upon Dox induction, while HCC in the clinic usually develops from a few spots and spreads during progression. This discrepancy in the approach of tumor expansion could cause problems in the translatability of the current study.

## 5. Conclusions

In conclusion, the current study confirmed that PH can promote *kras^G12V^*-induced HCC development in zebrafish and enhance neutrophil-mediated oxidative stresses in their livers. In addition, PH could enhance the deregulation of molecular factors that potentially contribute to HCC development including the Hippo-YAP regulator S100A1 and the ribosome biogenesis repressor FGF13 in zebrafish livers during *kras^G12V^*-induced HCC development. These factors could serve as therapeutic targets for developing intervention methods that are intended to apply to patients following PH to reduce HCC recurrence. Overall, the findings from the current study could improve the understanding of the relationship between PH-induced liver regeneration and HCC development, which may provide clues for developing novel therapeutic strategies.

## Data Availability

The data presented in this study are available on request from the corresponding author.

## Supplementary Materials

The following supporting information can be downloaded at: https://www.mdpi.com/article/10.3390/cancers16101793/s1, Figure S1: Uncropped images of S100A1 blots. (A) The image of the membrane of S100A1 blots under UV light. The membrane was cut into two strips. The upper strip was stained with the anti-β-actin antibody, while the lower strip was stained with the anti-S100A1 antibody. (B) The image of the membrane in (A) under white light; Table S1: List of primers used in RT-qPCR; Table S2: Genes up/downregulated in both *kras+* livers and PH livers; Table S3: Genes up/downregulated only in PH livers.
