# Supplementary material for: Partial Hepatectomy Promotes the Development of KRASG12V-Induced Hepatocellular Carcinoma in Zebrafish
Source: Cancers (Basel). 2024 May 8;16(10):1793. doi: 10.3390/cancers16101793 (PMC11119731; doi:10.3390/cancers16101793)
Supplement: Supplementary file 1 [file cancers-16-01793-s001.zip › cancers-2960391-supplementary.pdf]

cancers

MDPI

Article

Partial Hepatectomy Promotes the Development of KRASG12V-induced Hepatocellular Carcinoma in Zebrafish

Mingkai Zhu [1,2], Yan Li [1], Dong Liu [2,*] and Zhiyuan Gong [1,*]

Table S1. List of primers used in RT-qPCR.

Genes	Forward	Reverse
acta2	TGATGAAGCCGGACCCTCTA	AGAAGTGGGCAGTGCAAAGA
ccnb1	GGTGACTGGACCCCTACTCT	CCTCGTTGACCCTCACAACA
cdk1	CTCTGGGGACCCCTAACAAT	CGGATGTGTCATTGCTTGTC
col1a1b	TGAAATGAGGCGGCCAAAAC	TCCAGCATCCAGTTGACCAC
cybb	CCGGAGACTCAGGCTTTTGA	GTGATAGGATCGTCCTCCGC
fgf13b	AGAGTGGCATGGCGTTTCC	ATAGCCACCACTCGTAGCCC
hif1aa	GATGCTCGTCCACAGAAC	CACGTCGCAGACTTGATA
kras^{G12V}	CGACCACTACCAGCAGAACA	GCTTTTGCCTACGCCTACAG
lama5	CCACGGCCAAAGAAGAATGC	GCGTCACACCTACACTGACA
rps18	ATACAGCCAGGTCCTTGCTAATG	GTGACGGAGACCACGGTGAG
s100a1	CCTAATTCCCCCACACAGGT	TTATCCACCAGACGAGGAGCG
tgfb1b	ATAGCAGGTTTGTCCCGCAA	ATAGGAGCAAGAGCCAGTGC

Table S2. Genes up/downregulated in both *kras*+ livers and PH livers.

Genes Upregulated	PH vs Sham on Day 1		*kras*+ vs WT on Day 5	
**Gene ID**	**log2FoldChange**	**padj**	**log2FoldChange**	**padj**
fgf13b	5.794282	9.44E-15	4.651961	0.010768
serpinb1	2.556894	3.63E-07	4.677396	1.54E-30
LO018309.1	4.527712	0.00096065	2.839932	2.65E-05
asns	2.458471	0.00152875	1.079289	0.001419
BX571955.2	9.060113	0.00186814	11.93705	1.82E-10
ntd5	2.724144	0.00189110	6.869992	3.36E-34
s100a1	6.020058	0.00198862	3.340291	0.021678
ppp1r15b	4.322528	0.00486106	4.970812	8.99E-13
abcc6b.2	1.881603	0.00536820	1.434609	0.017899
osr1	8.69703	0.00781708	3.384821	0.045604
chst6	8.654082	0.00974658	4.171725	0.001213
si:ch211-286b4.4	2.234783	0.00974658	3.26067	0.0053
hpcal4	8.493526	0.01597101	6.463062	2.63E-14
cers2b	2.22468	0.02296315	1.466948	0.007234
stard10	1.711465	0.03280682	6.676752	1.32E-28
hmgn3	1.430703	0.0356808	1.576506	0.01595
**Genes Downregulated**	**PH vs Sham on Day 1**		***kras*+ vs WT on Day 5**	
**Gene ID**	**log2FoldChange**	**padj**	**log2FoldChange**	**padj**
cetp	-2.67926	4.03E-10	-3.28211	1.44E-11
cyp7a1	-3.36883	6.15E-10	-3.49177	0.02243
si:ch211-22d5.2	-6.67583	6.98E-09	-5.37469	0.008775

pebp1	-4.18151	1.49E-08	-6.45847	5.71E-32
mt2	-2.46864	2.62E-07	-3.94681	9.07E-13
zgc:172253	-2.26393	2.63E-06	-2.97349	2.02E-09
CU464134.1	-3.2615	4.00E-06	-7.11489	8.27E-19
igf2b	-1.8861	0.000154	-1.72091	0.015932
eepd1	-2.04567	0.000212	-2.73446	8.49E-05
eif4ebp3	-2.59437	0.000212	-4.26581	0.002446
myh7ba	-2.10363	0.000226	-4.71521	0.007172
hal	-1.81774	0.000246	-3.04578	6.64E-10
cd74a	-2.24291	0.000394	-1.55848	0.0142
pgam2	-2.97012	0.00141	-5.40401	9.29E-07
adh8a	-2.57928	0.002887	-6.67911	5.41E-32
cyp2x9	-2.79952	0.003349	-2.78846	7.71E-07
gtpbp4	-1.62187	0.003724	-2.19892	3.41E-05
caprin1b	-1.78551	0.0043	-1.40493	0.006724
cry1b	-1.54017	0.00453	-1.69201	0.025908
slc27a2a	-1.73248	0.005627	-5.56709	1.94E-24
slc16a7	-2.18387	0.005694	-6.76528	6.26E-13
abcc4	-2.29382	0.00588	-1.99992	0.003279
nudt17	-1.5891	0.006319	-1.4633	6.80E-05
cyp2k6	-2.93425	0.00941	-8.07478	2.32E-18
cyp2k22	-2.23549	0.014391	-7.11759	1.26E-07
fbp1b	-1.75086	0.015764	-5.86758	1.22E-21
rxraa	-2.33979	0.018463	-1.77409	0.028754
cyp2x12	-2.91969	0.018937	-6.41185	4.60E-11
pck1	-2.33748	0.019017	-6.62259	0.003295
ankrd24	-2.14075	0.019172	-3.75491	1.02E-05
zgc:153031	-1.90322	0.023104	-3.63714	4.16E-15
ddx21	-1.4548	0.024551	-2.10389	1.99E-05
si:ch73-288o11.5	-2.61601	0.02464	-3.65319	1.79E-07
rxrgb	-1.60002	0.028147	-2.55796	6.14E-14
hgd	-1.90664	0.028147	-5.10974	4.01E-19
cyp2n13	-1.94561	0.0309	-4.94566	5.80E-14
ahcy	-1.26898	0.032988	-3.50356	5.81E-22
slc47a1	-2.23125	0.032988	-3.78062	5.93E-07
cyp3a65	-1.77386	0.034457	-2.76754	1.92E-09
proca	-1.26949	0.035681	-0.904	0.045829
si:dkey-94e7.2	-3.17057	0.038219	-4.84254	1.22E-15
rsl24d1	-1.52859	0.04061	-2.12142	1.56E-05
apoba	-1.87765	0.046413	-4.87311	2.12E-06
ankrd9	-2.84836	0.047059	-5.04406	9.90E-05
got1	-1.56529	0.049467	-2.18536	0.000403

Table S3. Genes up/downregulated only in PH livers.

**Genes Upregulated**			**Genes Downregulated**		
**Gene ID**	**log2FoldChange**	**padj**	**Gene ID**	**log2Fold-Change**	**padj**
raver2	3.478661	5.50E-10	*crybg1b*	-5.96418	1.37E-22
tlr18	4.270187	6.98E-09	*cyp7a1*	-3.36883	6.15E-10

CU468730.1	7.678782	7.20E-05	*si:ch211-22d5.2*	-6.67583	6.98E-09
tnnt3b	9.90009	0.00019	*armc2*	-10.907	4.62E-08
BX511067.1	24.48908	0.000226	*si:dkeyp-73d8.9*	-29.4535	1.29E-06
sst1.2	3.284441	0.000226	*CU464134.1*	-3.2615	4.00E-06
si:ch211-270n8.1	2.023279	0.000298	*gnai2b*	-2.4356	6.06E-06
atp2a1l	9.524161	0.000336	*zgc:152753*	-2.40664	9.01E-06
cxcl8b.3	2.276303	0.001025	*pde5aa*	-10.5767	9.75E-06
fkbp2	1.939463	0.001187	*CR318601.1*	-3.59715	3.19E-05
cnpy1	2.085854	0.00141	*mhc2b*	-2.07675	8.34E-05
BX322792.1	9.088049	0.00182	*CU326366.2*	-9.97757	0.000226
mvda	3.679515	0.00182	*prkd4*	-3.55058	0.000311
si:dkeyp-107f9.2	8.989167	0.002371	*cd74a*	-2.24291	0.000394
si:dkey-228a15.1	1.927204	0.00255	*si:ch211-250k18.7*	-2.26293	0.00055
dbi	1.962446	0.00271	*pla2g4ab*	-9.32298	0.000582
wu:fd46c06	1.722508	0.00271	*pcdh1g18*	-22.8478	0.000984
si:dkey-105h12.2	1.847736	0.002782	*zgc:103700*	-1.98876	0.001025
hp	3.724809	0.003252	*CT867973.2*	-22.755	0.001025
anxa2a	1.570429	0.004061	*itgb5*	-2.252	0.001109
crp2	2.253436	0.004189	*si:ch211-181d7.1*	-4.0402	0.001669
CU570782.1	8.821518	0.005287	*cdc42bpab*	-3.25991	0.002118
sox4b	1.820116	0.00579	*cyp27b1*	-2.70079	0.002254
agr2	2.5585	0.005827	*ccl36.1*	-2.13146	0.00255
hmgcs1	3.479807	0.006045	*rec8b*	-8.91938	0.00255
npsn	2.313444	0.006045	*ctss2.2*	-2.5844	0.002582
hbl2	4.208621	0.007161	*mhc2a*	-3.50821	0.003349
AL845362.1	9.060736	0.007182	*si:dkey-11f4.20*	-3.0613	0.003398
acbd7	2.115806	0.007461	*anxa3b*	-1.6439	0.003466
ppib	1.51518	0.007848	*fblim1*	-2.14767	0.00353
si:ch211-214j24.15	3.114974	0.008314	*c1galt1a*	-8.80267	0.003906
sb:cb37	1.539925	0.008669	*fat2*	-5.45327	0.0043
krtcap2	1.515832	0.008884	*si:ch211-79k12.1*	-1.58443	0.00433
stk40	2.112158	0.009152	*thbs4b*	-3.35822	0.00453
acat2	2.200173	0.009556	*CU855930.2*	-8.78554	0.00453
CR626907.3	1.408087	0.009747	*cd74b*	-1.94742	0.005193
pvalb3	3.629958	0.010253	*cdo1*	-1.81995	0.00558
CABZ01061592.1	4.083059	0.010342	*si:dkey-9c18.3*	-9.41301	0.00558
postnb	2.102819	0.013176	*slc27a2a*	-1.73248	0.005627
si:zfos-1897c11.1	8.932785	0.014391	*CR753876.1*	-1.61026	0.006045
nrg2b	8.527183	0.014708	*si:ch211-217k17.11*	-1.97659	0.006906
alg5	1.563213	0.016356	*krt18a.2*	-2.00589	0.00704
mdkb	1.790627	0.016656	*zgc:194101*	-3.23023	0.007161
rpn1	1.363031	0.016656	*ctsbb*	-8.7534	0.007182
cyp3c4	2.158782	0.019001	*rrp1*	-1.89528	0.008669
zgc:123103	2.447353	0.019158	*cyp2k6*	-2.93425	0.00941
ost4	1.472512	0.022551	*si:busm1-194e12.11*	-2.14172	0.009726
cars1	1.830103	0.022933	*si:dkey-27d5.3*	-8.60835	0.0099
ebp	3.467681	0.022963	*blk*	-3.49857	0.012364

BX323564.1	3.226513	0.026413	*cxcl11.6*	-4.55238	0.012364
myhz1.1	5.348327	0.027891	*FP236331.1*	-2.44879	0.015941
slc1a4	4.021798	0.031494	*BX323596.2*	-3.52788	0.016751
CU469577.1	1.725836	0.031691	*CABZ01111959.1*	-8.46499	0.019001
si:dkey-183n20.15	1.867609	0.034457	*CABZ01073795.1*	-3.10266	0.019158
slc43a3b	1.866646	0.034457	*slc8a4b*	-5.56656	0.019158
si:ch211-212c13.8	1.564293	0.035681	*cacna1aa*	-8.59395	0.023104
lyve1a	2.403602	0.038219	*cyp2r1*	-1.65699	0.024871
rnd2	2.608239	0.04061	*plekhg6*	-8.32129	0.0309
CABZ01079192.1	2.004205	0.04061	*zfp36l1b*	-1.56347	0.032139
tmem263	1.370816	0.04061	*ptk2ab*	-1.61247	0.032807
chia.6	1.425481	0.041387	*loxl4*	-2.12842	0.034457
plppr5a	8.226123	0.045582	*irf1b*	-2.56474	0.035471
sinhcaf	1.424048	0.046413	*proca*	-1.26949	0.035681
nkx6.3	2.218194	0.047059	*epob*	-4.67739	0.035681
si:ch73-44m9.5	2.639637	0.049447	*zgc:194246*	-8.63239	0.035901
			dock11	-2.07097	0.04061
			si:dkey-28k24.2	-2.61835	0.04061
			cxcl11.6	-4.53957	0.045487
			tnikb	-1.71311	0.046006
			tspan2a	-3.72472	0.046381
			nitr12	-8.15367	0.046396
			zgc:152658	-8.1592	0.04689
			dus3l	-1.47694	0.04827
			zgc:174354	-8.20838	0.048961

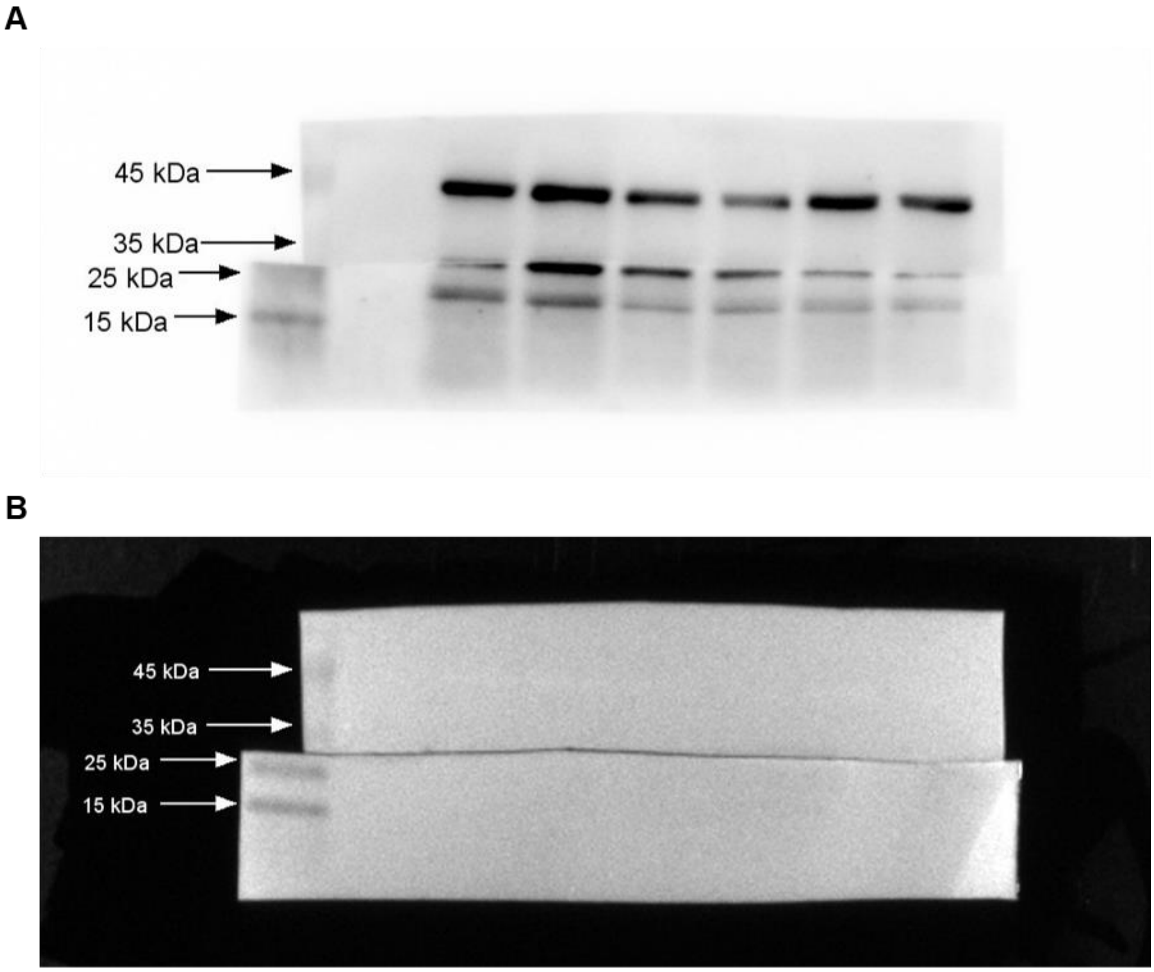

Figure S1. Uncropped images of S100A1 blots. (A) The image of the membrane of S100A1 blots under UV light. The membrane was cut into two strips. The upper strip was stained with the anti-β-actin antibody, while the lower strip was stained with the anti-S100A1 antibody. **(B)** The image of the membrane in **(A)** under white light.
